# Transcatheter Tricuspid Valve Replacement Beyond Boundaries

**DOI:** 10.1016/j.jaccas.2025.104353

**Published:** 2025-08-13

**Authors:** Robin Le Ruz, Isaac George, Vratika Agarwal, Tamim Nazif, Mark Lebehn, Carolina Pinheiro Rezende, Torsten P. Vahl, Martin B. Leon, Rebecca T. Hahn, Susheel K. Kodali

**Affiliations:** a Nantes Université, CHU Nantes, Interventional Cardiology Department, L'institut du thorax, Nantes, France; b Nantes Université, CHU Nantes, CNRS, INSERM, L'institut du thorax, Nantes, France; c Department of Medicine, Columbia University Irving Medical Center, New York, New York, USA

**Keywords:** heart failure, interventional cardiology, structural heart disease, tricuspid regurgitation

## Abstract

Transcatheter tricuspid valve replacement (TTVR) is now an available option to treat patients with severe symptomatic tricuspid regurgitation. Because of the early experience in this field, many challenges remain to be addressed. In this case series, we present 3 different situations that underline the difficulties in the clinical management of these patients. From a decision-making process standpoint, we discuss the relevance of TTVR in a patient presenting with an advanced cardiohepatic syndrome, as well as the case of a relatively young patient referred for tricuspid valve intervention following prior left-sided valves surgery. More from a technical and interventional strategy perspective, we describe a successful TTVR implantation after a failed transcatheter repair. TTVR has demonstrated convincing safety and efficacy results in large population samples. However, patient selection in clinical practice is still challenging, and this case series helps to refine our understanding of the role of TTVR in very specific clinical settings.

## Graphical Abstract

**Figure:**
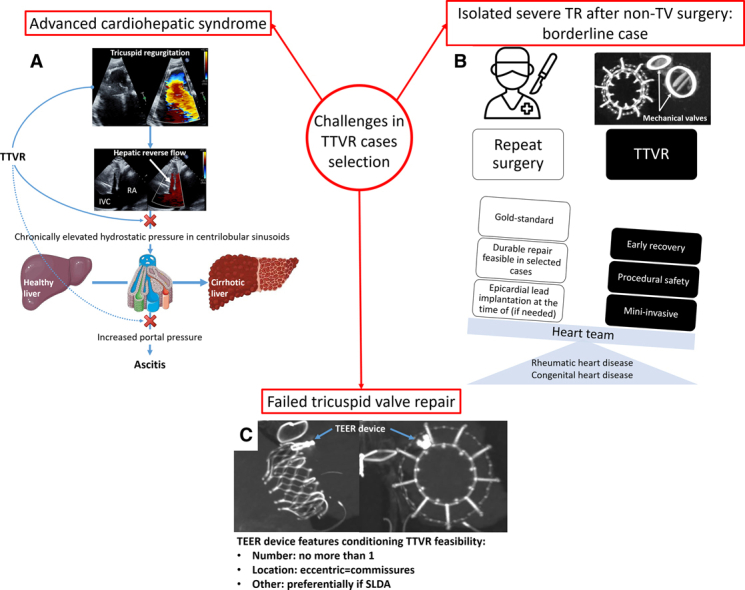

**Visual Summary:**
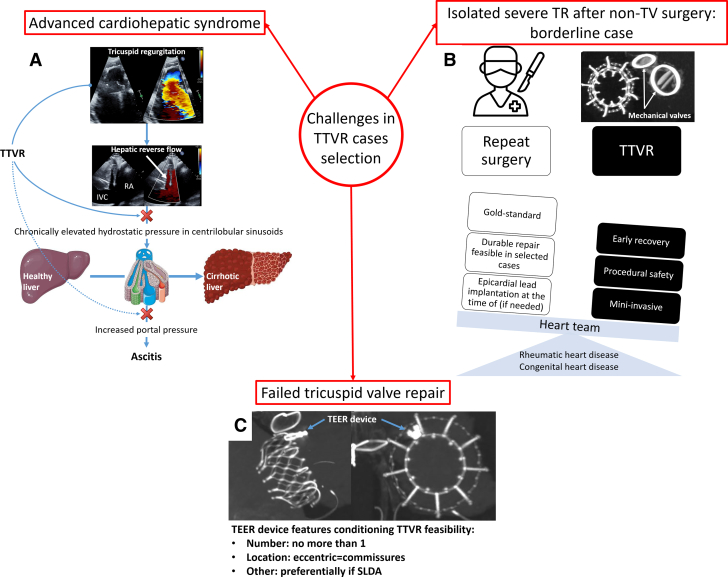
Illustration of the Main Features of the Clinical Challenges Described in This Case Series (A) Some aspects of the pathophysiology of cardiohepatic syndrome and the potential of transcatheter tricuspid valve replacement in these patients. (B) The heart team considerations in addressing isolated severe tricuspid regurgitation in high-risk patients with prior surgical treatment. (C) transcatheter tricuspid valve replacement successful implantation in a patient with failed transcatheter edge-to-edge repair. SLDA = single-leaflet device attachment; TEER = transcatheter edge-to-edge repair; TR = tricuspid regurgitation; TTVR = transcatheter tricuspid valve replacement; TV = tricuspid valve.

Transcatheter tricuspid valve replacement (TTVR) is a therapy now available in clinical practice. Given its recent emergence, many questions remain unanswered, and its role in some specific clinical scenarios has to be explored. The field of transcatheter intervention for tricuspid regurgitation (TR) is relatively new, and patients often present with many comorbidities and complex clinical histories. The distinctive profiles of these patients sometimes make the decision-making process and intervention more complex. We therefore sought to report a series of TTVR cases that underline some important features of the challenges faced in the management of these patients.

**Take-Home Messages**

• Cardiac cirrhosis patients with severe and symptomatic TR may be a subgroup population deriving great clinical benefit from TTVR.
• In patients with prior history of cardiac surgery presenting with significant isolated TR, TTVR appears to be a reliable treatment to avoid high-risk redo surgery.
• Certain cases of failed TEER could be treated with TTVR when appropriately selected in expert centers.

## Cases

### Patient 1

An 84-year-old man with known heart failure with preserved ejection fraction and permanent atrial fibrillation was admitted for altered mental status and lethargy. The initial work-up was consistent with hepatic encephalopathy related to a newly diagnosed cirrhosis (Child-Pugh class C). In addition, the patient presented with hypotension (89/53 mm Hg) and elevated lactic acid (up to 6.5 mmol/L). Transthoracic echocardiography was obtained revealing a severely dilated, mildly dysfunctional right ventricle and torrential TR with severely tethered leaflets. Based on the negative diagnostic laboratory work-up, his cardiac condition was considered as the main cause of cirrhosis. Initial medical management consisted of lactulose, diuretics, and dobutamine. Given his malnutrition status, he was rapidly started on nasogastric tube feeds. The patient was extremely frail and unable to get out of bed without assistance. Two key questions arose at the heart team meeting: 1) Was the TR responsible for his clinical picture? 2) Would fixing the TR reverse his clinical course? Palliative care was also part of the initial discussion because of the patient’s critical status at admission and the severe decompensated cirrhosis. With initial medical therapy, the patient's condition improved slightly with lactic acidosis resolving and mental status improving. The patient was sent to a rehabilitation facility to gain physical strength and improve nutritional status. The patient presented again 1 month later, and the decision was made to proceed with TTVR given the prohibitive risk for surgery and the anatomic ineligibility for transcatheter edge-to-edge repair (Video 1). The intervention was performed with a successful implantation of an EVOQUE valve system (Edwards Lifesciences). After 7 days, the patient was discharged with continuation of physical therapy sessions at home. Subsequent follow-up visits up to 6 months showed a continuous improvement in symptomatic status, resolution of abdominal distention and lower extremity edema (LEE), and major improvement in functional capacity: 6-minute walk test distance increased from 137 m after rehabilitation to 393 m at 6 months after TTVR (Table 1). In addition, cirrhosis severity was improved at last follow-up from Child-Pugh class C to A.

**Table 1:** Multimodality Evaluation of Patient 1 Evolution Before and After TTVR

	Baseline	Discharge	30 d	3 mo	6 mo
Heart failure medication					
Torsemide, mg	40	30	30	10^a^	10^a^
Spironolactone, mg	25	25	25	25	25
Dapagliflozin, mg	—	—	—	10	10
Laboratory results					
Bilirubin, total, mg/dL	4.2	2.5	2.3	1.6	—
Alkaline phosphatase, IU/L	212	201	225	198	—
GGT, U/L	244	300	—	—	—
AST, U/L	102	79	55	57	—
ALT, U/L	45	28	19	26	—
Cardiac imaging					
RVEDV, mL, CT-derived	302	—	194	—	—
RV/LV EDV ratio CT derived	4.5	—	2.1	—	—
Cardiac output by echocardiograph, L/min	5.7	7.2	6.1	—	—
IVC diameter, mm	40	28	30	—	—
Symptomatic and functional evaluation					
NYHA functional class	IV	—	II	I	I
6MWT	137	—	—	—	393

6MWT = 6-minute walk test; ALT = alanine transaminase; AST = aspartate aminotransferase; CT = computed tomography; GGT = γ-glutamyl transferase; IVC = inferior vena cava; RV/LV EDV ratio = right ventricle-to-left ventricle end-diastolic volume; RVEDV = Right ventricular end-diastolic volume; TTVR = transcatheter tricuspid valve replacement.

a As needed.

### Patient 2

A 66-year-old woman was admitted for worsening LEE and abdominal swelling. She had a complex prior history of rheumatic heart disease treated 17 years prior with mechanical aortic and mitral valve replacements along with a De Vega tricuspid valve (TV) annuloplasty. Based on investigations, the patient was found to have a torrential TR secondary to thickened and markedly tethered leaflets. Albeit redo surgery was initially considered for isolated TV replacement, the patient’s condition quickly declined, and she was hospitalized for volume overload. She required aggressive intravenous diuretics and repeated paracentesis. Her clinical condition continued to deteriorate, and she became extremely frail. She was seen by the surgeons and deemed to be a prohibitive surgical risk owing to frailty and chronic hepatopathy. In this setting, given the lack of improvement after heart failure optimization, it was decided to proceed with transcatheter tricuspid valve replacement with the Edwards EVOQUE valve, which was performed successfully with good-quality imaging despite the presence of mechanical valves (Video 2). Postinterventional course was notable for atrial fibrillation with rapid ventricular response, which was managed with amiodarone administration, and episodes of hypotension requiring pressors and inotropes; the patient was weaned off medications 6 days later. In addition, intravenous diuresis started 4 days before TTVR was maintained afterward and transitioned to oral treatment the day before discharge. Given some concerns regarding an ongoing slow bleed in the hypopharynx noticed before extubation, a heparin/warfarin bridge was delayed once it was confirmed that the hemorrhagic risk was controlled. The patient returned home 12 days after the procedure. At 1-year follow-up visit, she reported feeling well, with complete resolution of LEE and abdominal bloating without the need for any new paracentesis. More than 4 years after the intervention, the patient remained free from cardiovascular rehospitalization, with a normal functioning TTVR platform and without need for diuretics. However, she was admitted for hemorrhagic events on vitamin K antagonists (gastrointestinal, articular), all of which were managed conservatively.

### Patient 3

A 69-year-old woman with a complex medical history including atrial fibrillation, type A aortic dissection repaired 4 year earlier, and transcatheter edge-to-edge repair (TEER) for severe MR and TR 3 years earlier was seen in the clinic for a second opinion regarding management of heart failure. The valve repair seemed to have shown a durable and satisfactory MR reduction; however, torrential TR recurred shortly after the intervention due to the occurrence of single-leaflet device attachment. Because of worsening symptoms despite aggressive medical management, valve reintervention for the TR was considered. Despite her relatively young age, heart team evaluation determined the patient to be at high surgical risk owing to prior operations and frailty. In terms of transcatheter options, redo TEER was not considered because of a large coaptation gap and primary leaflet degeneration. Therefore, TTVR was considered the only possibility. The presence of a detached clip on the anterior leaflet posed challenges to implanting an EVOQUE valve, which relies on leaflets for anchoring. The concern was the clip would hinder the anchors from getting behind the leaflets as well as make imaging difficult. However, because the detached clip was close to the anteroseptal commissure, it was determined that it would leave enough space in the central portion of the valve to maneuver the delivery system and deploy the valve. TTVR was performed taking particular caution to locate and assess the position of the anchors relative to the TEER device (Video 3). Ultimately, anchors ended up landing on both sides of it, without any major spatial conflict, and no paravalvular leak. At 1-year follow-up the patient expressed the feeling of being “reborn”, without any further heart failure symptoms.

## Discussion

Individuals undergoing TR intervention are complex patients, and their management requires a global and multidisciplinary approach, as does any other patient with heart failure. TTVR now enables treatment of patients in a more advanced stage of their disease progression. Our case series depicts important aspects of the challenges we encounter in TTVR patient selection.

### Advanced cardiohepatic syndrome

Patient 1 reflects the vicious cycle that cirrhotic patients experience when combining severe TR and chronic liver disease, generating a pattern of congestive hepatic injury, superimposed on the primary etiology of the disease.^1^ In that setting, the resultant venous congestion causes structural damage on the centrilobular liver sinusoids and exacerbates the portal hypertension, provoking recurrent ascites. From a pathophysiologic standpoint, the existence of a hemodynamically significant hepatic venous pressure gradient in these patients makes them particularly vulnerable to any increase in central venous pressure (CVP).^2^ TR could therefore be a cause of decompensation in chronic hepatopathies. This is supported by the fact that CVP is recognized as the main predictor for the development of hepatic dysfunction in patients with chronic heart failure.^3^ In our case, because of the critical condition at the time of presentation, we could not predict that treating the valve disease would translate to the tremendous clinical benefit the patient would derive. Often, these patients are deemed too sick to benefit from the intervention, so-called cohort C. Given the above-mentioned interplay between liver disease and volume overload secondary to TR, chronic hepatic disease in TTVR candidates should not be a limiting factor per se, and dedicated studies are required to define the role of this therapy in this specific subgroup of patients.^4^ In fact, these patients may derive the greatest benefit from the restoration of a lower CVP after TR suppression. However, the etiology of the liver dysfunction might play an important role, and the patients with a primary cardiac cause might be those who improve the most after TTVR, as in the case described here.

### Isolated severe TR after non-TV surgical treatment

Patient 2 is representative of patients who have already undergone surgery, mostly for valvular disease, either related to left-sided valvulopathies or due to congenital right ventricular outflow tract dysfunction, and now present with symptomatic, severe isolated TR.^5^^,^^6^ In this case, the De Vega procedure, a suture annuloplasty technique that is no longer performed owing to unsatisfactory results, does not require the implantation of bioprosthetic material such as a ring or a stent frame that would consist of a reliable landing zone for balloon-expandable valve platforms. Regarding the benefit/risk balance, the prognostic impact of repeated cardiac surgeries is well known and is part of the cardiac surgical risk scores most frequently used in clinical practice (Society of Thoracic Surgeons and EuroSCORE II scores), albeit not included in TRI-SCORE, the most recent score developed specifically for patients with TR.^7^ In addition, these patients, particularly if they are relatively young, as frequently encountered in rheumatic and congenital heart diseases, may benefit from a lifetime management approach implementing a mini-invasive percutaneous treatment at some point in their clinical course, as a potential bridge to a later redo surgery. Given the safety profile of the TTVR procedure,^8^ this could become a reliable strategy in selected borderline patients evaluated for isolated severe TR with a prior history of cardiac surgery. Echocardiographic imaging in these cases may be challenging given the presence of surgical material, and this has to be carefully assessed during initial work-up. Intracardiac echocardiography might be needed intraprocedurally to overcome those difficulties.

### TTVR after failed TEER

TEER is currently the most studied, advanced, and mature therapy for the percutaneous treatment of TR. However, residual TR greater than moderate severity has also been found as a strong predictor for postprocedural clinical outcomes.^9^ Therefore, when considering patients for intervention, it should be taken into consideration whether adequate TR reduction can be achieved. One advantage of TTVR is that it has been shown to effectively suppress TR in most cases, with more than 90% of patients showing grade mild or less after the intervention.^8^ Our case demonstrates that TTVR is an option for significant residual TR after TEER. This obviously requires a meticulous anatomic assessment to determine the patient's eligibility for this kind of approach. The presence of more than 1 TEER device or a noncommissural location would be, in our opinion, 2 potentially significant limiting criteria for TTVR. In our case, because of the single-leaflet device attachment, the device landed in a more ventricular position allowing the TTVR platform to accurately seal into the native tricuspid annulus; thus, preventing the occurrence of any significant paravalvular leak. With the growing experience in the field, these cases may be performed safely;^10^ however, evaluation in highly specialized centers is mandatory to ensure procedural safety and success.

## Conclusions

As the TTVR field expands, our understanding of the role of this therapy will grow and enable better patient selection. The 3 successful patient stories reported here are a call for further studies to assess the reliability of the assumptions made based on these limited examples.

## Funding Support and Author Disclosures

Dr Le Ruz's research fellowship was funded by 10.13039/501100003100Fédération Française de Cardiologie, Groupement Interrégional de la Recherche Clinique et de l'Innovation Grand Ouest, Franco-American Fulbright program, Monahan foundation, Centre Hospitalo-Universitaire de Nantes, and Columbia University Irving Medical Center Interventional Cardiology Department. Dr George has received consulting fees from Cardiomech, Mitremedical, Atricure, Vdyne, Valcare Medical, DurVena, MITRx, and Johnson&Johnson. Dr Nazif has reported institutional funding to Columbia University Irving Medical Center from Medtronic, Boston Scientific, and Edwards Lifesciences and has received consulting fees from Medtronic, Boston Scientific, and Edwards Lifesciences. Dr Vahl has reported institutional funding to Columbia University Irving Medical Center from Boston Scientific, Edwards Lifesciences, JenaValve, and Medtronic and has received consulting fees from Abbott Vascular, 4C Medical, Innovheart, and Philips. Dr Hahn has received speaker fees from Abbott Structural, Baylis Medical, Edwards Lifesciences, Medtronic, Philips Healthcare, and Siemens Healthineers; has held institutional consulting contracts for which she receives no direct compensation from Abbott Structural, Edwards Lifesciences, Medtronic, and Novartis; and has been Chief Scientific Officer for the Echocardiography Core Laboratory at the Cardiovascular Research Foundation for multiple industry-sponsored tricuspid valve trials, for which she receives no direct industry compensation. Dr Kodali has received grant support, paid to his institution, from Medtronic, Boston Scientific, and Abbott Vascular; has received consulting fees from Abbott Vascular, Claret Medical, Admedus, and Meril Life Sciences; and holds equity options in BioTrace Medical, Dura Biotech, and Thubrikar Aortic Valve. All other authors have reported that they have no relationships relevant to the contents of this paper to disclose.

## Appendix

Video 1 Patient 1 Baseline Tricuspid Valve Imaging Transesophageal echocardiography imaging showing on color Doppler torrential tricuspid regurgitation with severely tethered leaflets.

Video 2 Patient 2 Intraprocedural Multiplanar Reconstruction Imaging Transesophageal echocardiography imaging demonstrating good visualization of the leaflets and their location relative to the anchors despite the presence of mechanical valves in the mitral and aortic position.

Video 3 Patient 3 Intraprocedural Anchor Check Transesophageal echocardiography depicting the close proximity between the TEER device (located at 5-o'clock position on blue plane), imaged in red box on the left side, and the anchors.
